# Molecular characterization of gliotoxin synthesis in a biofilm model of *Aspergillus fumigatus*

**DOI:** 10.1016/j.bioflm.2024.100238

**Published:** 2024-11-06

**Authors:** Alicia Gomez-Lopez, Candela Fernandez-Fernandez

**Affiliations:** a Mycology Reference and Research Laboratory, National Center for Microbiology (CNM-ISCIII), Madrid, Spain; b Center for Biomedical Research in Network in Infectious Diseases (CIBERINFEC-CB21/13/00105), Carlos III Health Institute (ISCIII), Madrid, Spain

**Keywords:** *Aspergillus fumigatus*, Biofilm, Secondary metabolism, Gliotoxin, Liquid chromatography, Transcriptional analysis, qPCR

## Abstract

Mycelial growth as biofilm structures and the activation of secondary metabolism leading to the release of low-molecular-weight molecules (known as secondary metabolites), are among the previously described strategies used by the filamentous fungi *Aspergillus fumigatus* to adapt and survive. Our study unveils that *A. fumigatus* strains can activate mechanisms linked to the production of gliotoxin, a crucial metabolite for *Aspergillus*, in the established *in vitro* biofilm model. Gliotoxin production exhibits strain- and time-dependent patterns and is associated -in a coordinated manner-with the expression levels of several genes involved in its regulation and synthesis. The transcriptional study of some of these genes by qPCR shows temporal inter-strain differences, which correlate with those obtained when evaluating the amounts of metabolites produced. Given that *A. fumigatus* forms biofilm structures within the site of infection, understanding the regulation of gliotoxin biosynthesis may have a role in the evolution of *Aspergillus* infection and guide diagnostic and treatment strategies.

## 1 Introduction

Different evasion strategies of *Aspergillus fumigatus* during its infectious process allows this fungus to adapt to unfavourable environmental conditions (nutrient scarcity, hypoxia, temperature, etc), avoid immune responses, and invade susceptible hosts. Biofilm formation and activation of secondary metabolism have been proposed as mechanisms involved in fungal pathogenesis [1].

The activation of secondary metabolism determines the production of secondary metabolites that promote fungal adaptation to the hostile conditions present not only in the environment but also within the host, protecting them from the action of the immune system and allowing the uptake of essential factors for fungal development. These are low-molecular-weight molecules that are not necessary for the exponential growth of the microorganism but favour its survival in adverse environments [2].

Fungal growth in the form of biofilm is recognized as an adaptive strategy. The structure of the biofilm is shaped by environmental conditions and affects the kinetics of secondary metabolite production [3] since these substances could function as cell communication signals even with other microorganisms during coexistence, contributing to the formation and maturation of the biofilm structure [4]. Studying these two processes simultaneously may offer clarifying results and establish theories related to the virulence of these strains.

One of the main and best characterized secondary metabolites in *A. fumigatus* is gliotoxin (GT), a mycotoxin included in the epipolythiodioxopiperazine (ETP) class. It can be detected in the serum of patients with invasive aspergillosis [1,5]. In a previous work, our group described different dynamics of GT and its inactive derivative bis(methylthio)gliotoxin (bmGT) production during *A. fumigatus* growth. The presence of GT may indicate an early phase of fungal development, whereas detection of bmGT may correspond to a more advanced stage of hypha maturation [6].

In *A. fumigatus*, as in other gliotoxin-producing fungi (*Gliocadium fimbriatum*, *Trichoderma* spp. or *Penicillium* spp.), the genes involved in the synthesis of this secondary metabolite are typically located next to each other in the genome and organized in contiguous gene clusters. The biosynthetic cluster for GT (*gli* BGC) includes several genes located on chromosome VI [7]. Different proteins govern the regulation of *gli* genes; thus, GT biosynthesis may be a hierarchical and multifactorial process.

Most BGCs fully characterized were those associated with metabolites (e.g., GT) produced in standard laboratory conditions (controlled media, temperature, planktonic status) [8]. It remains to be determined if BGC regulation differs under unusual growth conditions (such as during biofilm development) and whether the GT generating process in *A. fumigatus* under these conditions may provide relevant insights into how one process may influence the other. The molecular study of the cluster in a biofilm model will allow the identification of key targets involved in fungal development, adaptability, and invasion capacity. In this study, our objective was to determine whether the kinetics of GT production and the molecular mechanism involved follow a defined pattern, using two different clinical strains (from patients with invasive pulmonary aspergillosis) in an alternative *in vitro* growth model, a biofilm.

## 2 Materials and methods

### 2.1 *Aspergillus* spp. strains and culture conditions

For this study, one of the most studied *A. fumigatus* isolates, Af293 (ATCC MYA-4609, CBS 101355) type strain isolated from a patient with invasive aspergillosis [9] and one clinical strain (CM9160) isolated from an immunosuppressed patient with pulmonary aspergillosis (deposited in collection of the Mycology Reference and Research Laboratory, MRRL, Madrid, Spain) were used. The strains were grown on potato dextrose agar medium (OXOID, Madrid, Spain) at 30 °C.

### 2.2 Biofilm formation and characterization

We initially studied the capacity of the selected strains to form a biofilm in a 96-well microdilution plate format. A conidial suspension (size, 2x10^4^ cfu/mL) was dispensed into 96-well microplates (Costar® Corning Incorporated, Madrid, Spain) and grown statically for 24, 48, or 72 h at 37 °C in RPMI 1640 medium at pH 7, buffered with MOPS and supplemented with 2 % glucose (RPMI-G).

For biofilm characterization, biomass was assessed as described elsewhere, with modifications [10]. Briefly, medium was removed from each well at the fixed time points (24, 48 and 72 h, with special care not to disturb the biofilm structure during the process) and the mycelia allowed to air dry (30–120 min), stained with 0.2 % (w/v) crystal violet for 15 min at room temperature (RT), and next, rinsed with distilled water. The bound dye was extracted with 0.25 mL of 95 % ethanol and the amount measured at *λ*_620_ nm (OD 620) using a microtiter plate reader (ELISA EZ Read 400, Biochrom™) to determine the level of staining, which is proportional to the amount of biomass produced. Biofilm characterization was also assessed by measuring metabolically active cells using XTT incubation (indirect marker of metabolically active cells). At each fixed time, the medium was removed, and mycelia washed twice with PBS and next, XTT-menadione solution in PBS was added (0.05 mL, final concentration 0.113 mg/mL and 0.015 mg/mL, respectively).

The plates were incubated in dark at 37 °C for 2 h and then read at *λ*_450_ nm (OD 450) (ELISA EZ Read 400, BiochromTM) to determine the level of staining, which is proportional to the amount of metabolically active cells.

Expression analysis of the genes involved in gliotoxin biosynthesis by real-time PCR (qPCR)

To carry out the transcriptomic characterization of genes involved in gliotoxin synthesis, 5x10^5^ cfu/mL were adjusted in 20 mL RPMI-G medium. The plates (90 cm) were incubated at 37 °C at four fixed times (16 h, 24 h, 48 h and 72 h).

### 2.3 RNA extraction and complementary DNA (cDNA) synthesis

Once the established incubation times were completed, the formed biofilms were transferred to 50 mL conical bottom tubes (Falcon® Corning Science) and centrifuged at 3.500 rpm for 15 min. After completing supernatant removal, total RNA was isolated using TRI Reagent (Sigma-Aldrich®) according to the manufacturer's instructions. The mixture was resuspended and immediately transferred to a 2 mL tube containing 0.45–0.55 mm diameter Zyrconia beads (RiboPure™ Yeast RNA Purification Kit, Thermo Fisher Scientific, Madrid, Spain). Samples were subjected to eight 20 s cycles at 4.0 m/s in FastPrep (MP Biomedicals™), with 1-min breaks on ice to promote mycelial fragmentation.

After the mechanical breakage of mycelium, samples were incubated 5 min at RT to allow complete dissociation of the nucleoprotein complex; next, 0.2 mL of chilled chloroform was added to precipitate cell debris, manually shaken for 15 s, and incubated 2–3 min at RT before centrifugation (10 min, 12.000×*g*, 4 °C). The aqueous phase was transferred to a sterile tube, and 0.5 mL of isopropanol added for RNA precipitation. After incubating 10 min at RT, samples were centrifuged (10 min, 12.000×*g*, 4 °C) and the supernatant discarded. The resultant RNA was washed with 1 mL of 75 % EtOH and then removed by centrifugation (5 min; 7.500×*g*, 4 °C). Finally, the samples were allowed to dry for 5–10 min at RT to completely remove the ethanol by evaporation before reconstitution with 0.03–0.05 mL of RNAse-free water (DNA-free™ DNA Removal Kit, Thermo Fisher Scientific, Madrid, Spain). The amount of RNA extracted from each sample was quantified with Nanodrop One (Thermo Fisher Scientific, Madrid, Spain) and stored at −80 °C. Three to six replicates of each condition were prepared.

To remove contaminating DNA before the synthesis of complementary DNA (cDNA), samples were treated using the DNA-free™ DNA Removal Kit (Thermo Fisher Scientific, Madrid, Spain) following the manufacturer's instructions.

For cDNA synthesis, the iScript™ cDNA Synthesis Kit (Bio-Rad Laboratories, Inc., Madrid, Spain) was used, following the manufacturer's instructions.

### 2.4 Real-time PCR

LightCycler 480 II thermocycler (Roche Diagnostics®, Barcelona, Spain) was used for real-time quantitative PCR (qPCR). Seven genes (*gliK, gliP, gliT, gliI, gliG, gliC*, and *gliZ*) from the *gli* BGC were selected. The translation elongation factor 1 alpha*, tef1a* gene was used as the housekeeping gene [11] (Table 1). For the *gliK, gliP, gliT*, and *gliZ* genes, the primers described by *Kong* et al. were used [12], while the oligonucleotides used to amplify the *gliG, gliC*, and *gliI* genes were designed with the NIH Primer-BLAST oligo design program [13] using the sequence accessible for each gene in the *A. fumigatus*-specific database at the Broad Institute AspGD [14].

**Table 1:** Sequences of primers used in the study of gene expression. A brief description on activity of proteins encoded is also included.

Target Gen	Protein encoded/activity	Primer Type	Primer sequences (5’ → 3′)	size
*gliK*	GliK: gamma-glutamyl acyltransferase	Sense	ATGCCTCGATCCTCGACAAG	128 bp
		Antisense	CGAGATGAGGCCCAGGTAG	
*gliP*	GliP:Non-ribosomal peptide synthetase (NRPS)	Sense	CCGATGATGAAGAGACCACA	158 bp
		Antisense	AAGAGCAGCAAGGGAGTTTC	
*gliT*	GliT: thioredoxin reductase	Sense	ACGGAGGGCTTTTTGGTGT	168 bp
		Antisense	CTTTAACGGCGTGGCACAAT	
*gliZ*	GliZ: C6 zinc finger domain regulatory protein	Sense	CAGACCCTCAGCAGCCTAAC	113 bp
		Antisense	GGACAGTGGGAACAGTGGTA	
*gliI*	GliI: Aminotransferase	Sense	ATCAAGGCCATCCTCGTGTG	161 bp
		Antisense	GTGAAGGCGGTTTGCTCATC	
*gliC*	GliC: Cytochrome P450 oxidoreductase	Sense	TTGGTTCAAGTCACGGGGAG	169 bp
		Antisense	GTAGAGCGATTCGTCGAGGG	
*gliG*	GliG:Glutathione S-transferase	Sense	GAAACTGCGCAGCAACATTA	176 bp
		Antisense	TTGGCCATTTCTCAAACTCC	
*tef11a*	TeF1a: Translation elongation factor 1 housekeeping gene	Sense	CCATGTGTGTCGAGTCCTTC	152 bp
		Antisense	GAACGTACAGCAACAGTCTGG	

The reaction mix (final volume of 0.02 mL) was composed of 1x Sso7Advanced™ Universal SYBR® Green Supermix (Bio-Rad Laboratories, Madrid, Spain), 0.5 μM sense and antisense oligonucleotides, and 50 ng of cDNA. The mix included all the components necessary to carry out the amplification reaction (Sso7d fusion polymerase, dNTPs, MgCl_2_, etc.) and quantification of the formed product (SYBR® Green I Dye). The protocol used for Real-Time PCR consisted of a 30 s denaturation cycle at 95 °C, followed by 40 cycles of amplification and quantification of 15 s at 95 °C and 62 °C for 1 min (banding/extension), with a fluorescence reading; the final step was done at 95 °C and continuous fluorescence measurements (melting curves).

### 2.5 Expression analysis

Fold changes in expression were calculated using the 2^-ΔCt^ method for individual time points (16, 24, 48 and 72 h) and normalized to *tef1a* housekeeping gene. Each experiment was repeated at least three times, and all the gene expression values were used for comparison and graphical representation. Differences in expression values of each gene were calculated and represented on a logarithmic scale with respect to the expression values at 24 h (basal). Values equal to 1 (0 in the logarithmic scale) resulted in similar expression. Values < 1 (<0 in the logarithmic scale) indicated decreased expression of the gene in the conditions analysed, and values > 1(>0 in the logarithmic scale), increased expression.

### 2.6 Quantification of secondary metabolites with ultra-performance liquid chromatography

The quantification of diffusible secondary metabolites by liquid chromatography was performed using the supernatant collected at the same time points established before (16, 24, 48 and 72 h). For each time point, 0.2 mL of separated supernatant were treated with 0.4 mL of acetonitrile (AcN, PanReac Applichem®, Madrid, Spain), vortexed, and centrifuged (11.800×*g*, 10 min). The resulting supernatant was transferred to a new tube, and 0.4 mL of chloroform (Honeywell®, Madrid, Spain) added. After vortexing, the tubes were centrifuged (11.800×*g*, 5 min) to promote the incorporation of GT and bmGT into the chloroform organic phase; this resulted in three distinct phases from which the clear chloroform fraction was transferred to a new tube. The chloroform extraction was repeated two more times. The three clear extracts were collected in one glass tube and evaporated to dry residue (Concentrator plus, Vacufuge® plus; D-HV program, 70 min, 60 °C). The dry residue was resuspended in 0.1 mL of an AcN/H_2_O mixture (53:47), vortexed, filtered, and transferred to glass vials for chromatographic analysis. The samples were analysed by a reversed-phase liquid chromatographic method using the ACQUITY H-class system and a UPLC® HSS, C_18_, 1.8 μm, 2.1 × 75 mm column (Waters Chromatography®, Barcelona, Spain). Chromatographic separation was achieved by a gradient method in successive steps with a constant flow rate of 0.1 mL/min. A mixture of AcN and MilliQ-quality water was used as the mobile phase; the proportions of AcN and MilliQ-quality water are modified during the process, with the AcN proportion increasing (from 40 % to 65 %) in the initial steps of the gradient to first elute the more non-polar component (bmGT) from the mixture. The use of a gradient that alternates between solvents with polar and non-polar properties facilitates the separation of the two molecules. The quantification was done using an ACQUITY UPLC Photodiode Array (PDA) detector (Waters®) that allows specific detection and quantification of low concentrations of analytes from the analysed samples, as well as the establishment of specific UV profiles. This facilitates the identification of components of a similar chemical nature and difficult resolution with conventional HPLC methods.

The concentration of GT and bmGT, measured by chromatographic peak area, was previously standardized by evaluating the detector signal: peak area vs. standard GT/bmGT (Enzo biochem) concentrations over an analytical range of 0.25–32 mg/L, dissolved in AcN/H_2_O (53:47 v/v).

### 2.7 Statistical analysis

All experiments were performed in triplicate. Values were expressed as the mean ± standard deviation. Statistical significance between groups was determined by a two-tailed analysis of the means (Anova) and Tukey's multiple comparisons test using the GraphPad Prism software version 9.0.2 (GraphPad Inc., La Jolla, CA, USA). A *P* value < 0.05 was statistically significant.

## 3 Results

### 3.1 Biofilm characterization

**Fig. 1:**
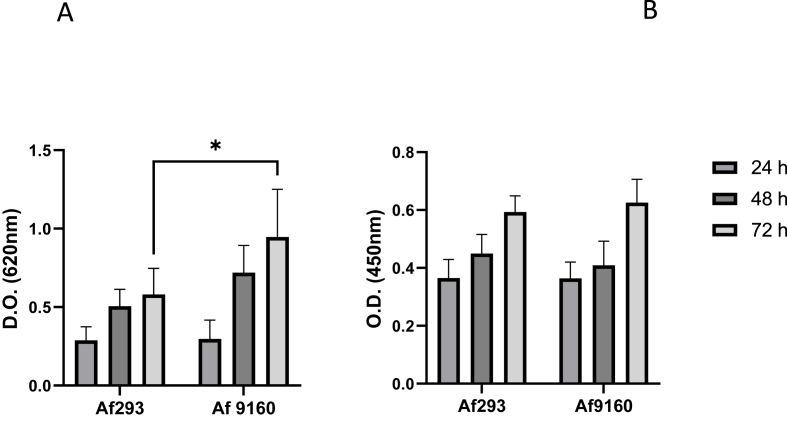
A) Biomass content of the biofilms at different times point measured by the CV assay (OD 620 nm). B) Cell viability of biofilms at different times point determined by the XTT assay (OD 450nm).

The two strains evaluated developed biofilm *in vitro*. In both cases, biomass and cellular activity increase over time. No significant differences in cellular activity were found between the strains, although the Af 293 isolate formed a less dense biofilm (lower biomass) (Fig. 1A, T = 72 h, OD 0.5 vs 0.9, *P* < 0.01).

### 3.2 Kinetics of secondary metabolites production

**Fig. 2:**
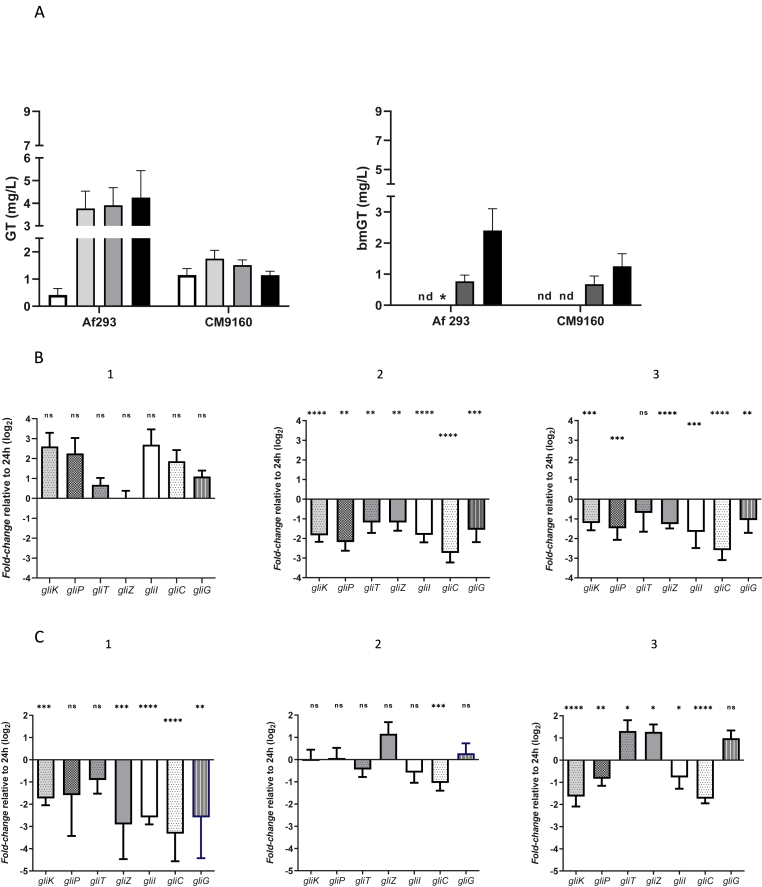
A) Gliotoxin (GT) and bismethylthiogliotoxin (bmGT) concentration detected in supernatant samples collected from biofilms grown at different incubation times. The mean value and the sd corresponding to three independent experiments are shown. (∗) concentration below the limit of quantification of the technique. (nd) no chromatographic peak area detected. B) Relative fold changes of the mRNA expression levels of studied gene involved in GT synthesis for Af 9160 strain (EP) at 16 (1), 48 (2) and 72 (3) h of incubation. C) Relative fold changes of the mRNA expression levels of studied gene involved in GT synthesis for Af293 strain (LP) at 16 (1), 48 (2) and 72 (3) h of incubation. (ns) statistically non-significant differences (*P* > 0.05). (∗) *P* < 0.05 (∗∗) *P* < 0.005 (∗∗∗) *P* < 0.0005 (∗∗∗∗) *P* < 0.0001.

The release of secondary metabolites followed a time and strain dependent pattern. GT was mainly detected in early phases (16 h–24 h), whereas bmGT appeared in more advanced stages of growth (Fig. 2A). A different pattern of production was seen depending on the strain. Quantitatively, Af 293 produced larger quantities of GT although CM9160 appears to generate more GT at shorter incubation times (early producer, EP). For this strain, a higher amount of GT was detected compared to Af 293 during the first 16 h of biofilm development, reaching a maximum peak at 24 h, followed by a subsequent decrease (a downward pattern at 48 and 72 h). On the contrary, minimum GT concentrations were observed for strain Af293 at 16 h, followed by an abrupt increase at 24 h and a continuing upward trend over time, defining it as a "late producer, LP”.

Both strains showed similar bmGT production pattern, with absence of this metabolite at shorter incubation times (16 h and 24 h) and increased production later (48 h and 72 h). For both strains, bmGT was absent at 16 h. Af 293 also produced higher amounts of bmGT than CM9160.

### 3.3 Expression analysis of the genes involved in the biosynthesis of GT

Transcriptional analysis also revealed inter-strain differences based on the temporal dynamics of GT production.

Of note, four genes *gliK*, *gliP*, *gliI*, and *gliC*, appeared up-regulated at early phase (16 h) when comparing to basal condition (24 h) for the EP strain (CM 9160; Fig. 2B1). A moderate increase in the relative expression of the mentioned genes (1.5-3-fold, *P* > 0.05) was observed. This activation of the biosynthetic machinery in the early stages of biofilm development could explain the higher GT amounts detected for this strain at this time point (1.14 vs 0.43 mg/L, Fig. 2A) and would justify the so-called EP we have assigned to this strain in relation to the other. Lower levels of relative expression of all genes were also observed thereafter (48 h and 72 h of biofilm development), coinciding with a downward pattern of GT production detected for this strain (Fig. 2B2 and 2B3, respectively).

On the other hand, biosynthetic genes appear downregulated in the LP strain at early time points comparing to basal condition (Af 293, Fig. 2C1). Additionally, the regulatory mechanism, encoded by the *gliZ* gene, appears overexpressed (48 and 72 h, Fig. 2C2 and 2C3), coinciding with a sustained increase of GT release for this strain (Fig. 2A). Due to the high variability found in the replicates, there were hardly any statistically significant differences in the degree of gene expression between 24 h and 48 h of incubation (Fig. 2C2). At 72 h, more genes of the biosynthetic machinery (*gliT*, *gliG*, Fig. 2C3) appear to be upregulated compared to the established baseline condition, coinciding with a later onset of GT production that increases over time in this strain (from 0.41 to 4.25 mg/L, Fig. 2A).

## 4 Discussion

Interest of *A. fumigatus* biofilm has begun to emerge in recent years and “omics” analysis serves as a major tool in investigating this growth form highly relevant to infection. At the site of infection, *A. fumigatus* forms biofilms that can facilitate its development and evade immune defence or the action of antifungal agents. Hence the importance of defining the mechanisms involved in the regulation of this common form of growth.

Our study shows that *A. fumigatus* strains differentially activate mechanisms associated with the production of certain secondary metabolites during biofilm development, even though the structure of the biofilm generated does not differ significantly among the studied strains. We hypothesized that during biofilm maturation *A. fumigatus* must trigger specific responses to adapt to hostile environments, and it is likely that this response is the result of complex, interrelated cellular mechanisms that vary between isolates. Among the wide variety of secondary metabolites produced by *A. fumigatus*, we focused on the kinetics of GT and bmGT production, due to their significant effect on pathological processes in humans and animals (they are involved in impairing the host immune system) and the potential role as a diagnostic biomarker [[15], [16], [17], [18]]. Previous studies elucidated that a specific group of genes in the *gli* BGC, appear expressed in a co-regulated manner, and are responsible for the biosynthesis of GT, among these genes, *gliP* or *gliZ* were found pivotal for GT production [19,20]. Interestingly, Owens et al. also described that members of this *gli* BGC showed increased abundance during biofilm growth [21].

In the present study, we found that GT is present in the supernatants of both studied strains, although with a different production pattern (time- and species-dependent). In a previous work, our group already described the differential dynamics in the production of these two metabolites, GT and bmGT, during the *A. fumigatus* growth [6]. In this study, we have also linked the dynamics of GT production to the differential molecular activation of the associated biosynthetic machinery. Considering study results, we have defined two differentiated phenotypic patterns between the two strains: the EP, characterized by the overexpression of several biosynthetic genes in the cluster (primarily *gliK*, *gliP*, *gliI*) during the initial stages of biofilm growth and the LP exhibiting down-regulation of these genes at the same times points. On the contrary, an analysis carried out at longer times of biofilm development (T = 72 h) unveils that the LP phenotype is associated to a genetic profile of increased expression of regulatory gene first, *gliZ*, and biosynthetic genes later, particularly *gliT*. This demonstrates a differential activation in two strains that produce similar biofilms (LP exhibited a less dense biofilm but with the same cellular activity) and associated with the same pathology.

The transcription factor GliZ, encoded within the cluster (by *gliZ*), has been proposed to act as a positive transcriptional regulator that controls the expression of enzymes involved in GT synthesis [19]. Moreover, replacement of *gliZ* (Δ*gliZ*) resulted in no detectable GT production and loss gene expression in the cluster, while placement of multiple copies of *gliZ* in the genome showed an increase of GT production.

Besides *gliZ*, other genes encode proteins involved in the different stages of GT formation from L-Phe and L-Ser: GliP (non-ribosomal peptide synthetase), GliT (thioredoxin reductase), GliG (glutathione S-transferase), or GliA (membrane transporter). Among them, *gliP* is the largest and codes for a non-ribosomal peptide synthetase, which catalyses the first step of GT biosynthesis by conjugating L-Phe and L-Ser. Under the same growth condition, we observed distinct expression patterns of *gliP* in the two strains studied. The expression of this gene, which is associated with the initial step in GT synthesis, is increased in the EP strain, which seems to activate the biosynthetic machinery earlier, compared to the LP strain that appears to activate it later. The differences in expression found in regulatory genes and in genes more directly involved in the biosynthetic process of GT allow us to hypothesize significant differences in cellular mechanisms related to their pathogenic capacity in two clinical isolates of *A. fumigatus* associated with the same pathology. It is important to note that in this study, we have not assessed the genetic variability of any of the genes in the cluster, and this variability could also be the cause of the different response observed regarding GT production. Previous work has demonstrated that secondary metabolite gene clusters, like the metabolites that they produce, are highly divergent between fungal species and across strains of a single fungal species [22,23].

Of note, bmGT was also detected under the growth condition stablished in the study. Increased levels of bmGT seem to correlate with a decrease of GT. There were differences in the production of this metabolite, i.e., the isolate that produced larger amounts of GT (LP, Af293) coincides with the greatest producer of bmGT. Previous studies show that S-adenosyl-l-methionine-dependent GT bis-thiomethyltransferase (GtmA), whose gene is not located in *gli* BGC, participates in the conversion of GT to bmGT, a negative regulator of GT biosynthesis, to protect *Aspergillus* against GT cytotoxicity [17,24].

Unfortunately, the expression of the gene encoding this methyltransferase was not an objective of our study, which specifically focused on the expression of some of the genes within the *gli* BGC. Therefore, we can conclude little about the generation of the GT derivative and the molecular pattern that governs it.

A limitation to this study is that includes only two isolates of A*. fumigatus* associated with the same pathology. However, it adds new information on differential GT production by clinical isolates and its impact as a virulence factor. In previous work, Kupfahl et al. described a trend towards the production of higher GT concentrations in strains from patients who experienced invasive aspergillosis compared to those that were merely colonizers [25]. Our research group is currently investigating isolates from other origins (cystic fibrosis, invasive aspergillosis) with alternative growth patterns, anticipating a divergence in behaviour concerning biofilm formation, activation of secondary metabolism and virulence. Further studies will also be necessary to determine the effect of genetic variability within the cluster and its relationship with the differential production of GT in different strains.

In summary, we found distinct phenotypes related to the kinetics of secondary metabolite production in two clinical strains of *A. fumigatus* (Af293 and CM9160). These phenotypes correspond to different patterns of activation of the associated molecular machinery. These differences may influence the pathogenesis, response to treatment, evasion of the immune response, invasiveness, etc. The expression of the regulatory gene *gliZ* firstly determines the level of GT production, constituting a possible target for the modulation of secondary metabolism and the response of the fungus to hostile conditions.

Using an *in vitro* model of biofilm of *A. fumigatus* this study shows the ability of the fungus to adapt metabolically to adverse conditions by activating secondary metabolism. The relevance of this metabolism for fungal survival makes it an interesting target in the development of new diagnostic and treatment tools.

## CRediT authorship contribution statement

**Alicia Gomez-Lopez:** Writing – review & editing, Writing – original draft, Validation, Supervision, Software, Resources, Methodology, Funding acquisition, Formal analysis, Data curation, Conceptualization. **Candela Fernandez-Fernandez:** Writing – original draft, Software, Methodology, Data curation.

## Funding sources

This work was supported by the Carlos III Health Institute ISCIII (Research Project AESi PI21CIII/00012, MPY 435/21; Government of Spain). AGL belongs to Center for Biomedical Research in Network in Infectious Diseases (CIBERINFEC-CB21/13/00105), ISCIII, Madrid, Spain.

## Declaration of competing interest

The authors declare that they have no known competing financial interests or personal relationships that could have appeared to influence the work reported in this paper.

## Data Availability

Data will be made available on request.
